# Methodological issues on ‘challenges and opportunities towards the road of universal health coverage (UHC) in Nepal: a systematic review’

**DOI:** 10.1186/s13690-020-00418-x

**Published:** 2020-04-29

**Authors:** Chhabi Lal Ranabhat, Devaraj Acharya

**Affiliations:** 1 Manmohan Memorial Institute of Health Sciences, Kathmandu, Solteemod Nepal; 2 Global Center for Research and Development (GCRD), Banasthali, Kathmandu, Nepal; 3 Bhairahawa Multiple Campus (Tribhuwan University), Siddharthanagar Rupandehi, Nepal

**Keywords:** Universal health coverage, Systematic review, Data base, Policy review

## Abstract

Challenges and opportunities towards the road of universal health coverage (UHC) in Nepal: a systematic review’ is a policy review paper and we published in BMC – Archives of Public Health. Policy research is the process of conducting research, analysis of, a fundamental social problem in order to provide policymakers with pragmatic, action-oriented recommendations for alleviating the problem. The objective of this paper is to illustrate some methodological issues used in that paper.

To the editor,

**Context**

Nepal’s commitment to universal health coverage (UHC) offers a starting point for rethinking the purpose and organization of the health system and an opportunity to introduce the quality of care agenda into policy discourse [1]. By constitution, there are three level of government with specific role: federal government makes policy, provincial government monitor the programs and local government implement health care services [2]. There are limited human resources in government health system [3], geographical difficulties [4], inequality in health service [3, 5], varieties of health care practice [6], limited numbers of research in health program and policy [7], there is no sufficient access for international journals and data base for researcher [8] and limited health budget in research [9] in Nepal. In this line, geographical difficulties to implement health related program, level of education of country, quality of research etc., health related to action research are limited and it is not possible to do research in full fledge way consedering publication and language bias.

It is well known that the paper ‘Challenges and opportunities towards the road of universal health coverage (UHC) in Nepal: a systematic review’ is primarily focused for Nepal. The objective of study was to report opportunities and possible challenges rather than number of people participated in the studies and types of studies. The policies consist of mostly, legal provision, standards, guidelines, programs and selected studies. In introduction part, there is well described about the health status of Nepal, search engines, strategies, major key words, inclusion and exclusion criteria and clear Preferred Reporting Items for Systematic Reviews and Meta-Analyses (PRISMA) follow chart have been described in methodology [10]. Likewise, results have been presented with 32 literatures in four categories and situations have been compared with other countries in discussion section. Such components are enough for explorative systematic review. The scope of this paper is quite limited and used resources are inevitable for English and some sorts of Nepali language and there is no possibility to use other references available in other language. Indirectly, it is the monitoring regarding the UHC situation of Nepal and resources are mostly available in PubMed, Google Scholar, Health Inter-Network Access to Research Initiative (HINARI), web pages of Nepal Governments and Google. All gray materials indexed in other sources are linked in Google and it is sufficient to cite. Indexed literatures are used, if they are extracted from available data base and not necessarily mentioned if they were not received. Some data bases have special characteristics for example, Cochrane library is popular for control trials, Embase is for biomedical and pharmaceutical and PsycoInfo is psychological research other than policy and program research. The systematic review is simply review with systematic way but it is not rocket science and available materials need to investigate carefully without bias but for the meta-analysis, there is special procedure and such format need to follow specially in clinical cases. This is a policy and program review and does not follow criteria of other articles like exploration of diseases prevalence, odds ratio and to be rigid with participants, intervention, comparison, and outcomes (PICO) strategies. This paper is more applicable for policy designers, program implementers and government authorities who are more responsible to achieve universal health coverage (UHC) till 2030 and similar countries.

**Application of systematic review with creative way**

Systematic review is a process of presenting previous research/data/evidence in a systematic way. It needs searching strategy of literatures, inclusion and exclusion criteria, number of included and excluded literatures, reasons for excluding and total used sources in paper. Usually, systematic review and meta-analysis is used together specially in epidemiological and clinical research [11] because those researches are more technical e.g. types of study (mostly, cross-sectional, case control and randomized control trial) and analysis of prevalence, odds ratio and PICO questions. It should be keep in mind specially for the young researchers that there is different between systematic review (SR) and meta-analysis and SR is possible without meta-analysis [12]. More importantly, implementation and action research does not necessarily to follow the reporting strategies of PRISMA guidelines. Such types of research are program design, implementation, evaluations, related policies, strategies, activities, etc. [13–15] Last but not least, SR must be used by creative model other than narrow and classical way so that coming researcher shall develop and improve with more pragmatic approaches.

**Application of study tools**

To assess the situation of universal health coverage in Nepal it is an explorative study, the findings have been presented in narrative way. Policy and program research are quite special and more contextual and they are liberal with methodological hegemony [16] . So, it is not pure quantitative, qualitative and combine study. The Mixed Methods Appraisal Tool (MMAT) is a critical analysis tool that is designed for the appraisal stage of systematic mixed studies; qualitative, quantitative and mixed methods [17]. MMAT compiles some quantitative and some qualitative studies. It is possible for the qualitative data to be converted into quantitative data during and/or in preparation for analysis (or vice versa) [18]. In policy review, such tool is not useful and narrative analysis is the best way. Despite the context, nature of study, data reporting and synthesis, we would clearly mention the limitation of study. Nonetheless, to prepare specific systematic review protocol, complete the review process, providing specific comments (if any) by editor and approve the manuscript have the sole right with journal.

## Data Availability

Not applicable.

## Abbreviations

HINARI: Health Inter-Network Access to Research Initiative
MMAT: Mixed Methods Appraisal Tool
PICO: Participants, Intervention, Comparison, and Outcomes
PRISMA: Preferred Reporting Items for Systematic Reviews and Meta-Analyses
SR: Systematic review
UHC: Universal health coverage
